# Molecular Characterization and Enological Potential of A High Lactic Acid-Producing *Lachancea thermotolerans* Vineyard Strain

**DOI:** 10.3390/foods9050595

**Published:** 2020-05-07

**Authors:** Georgios Sgouros, Athanasios Mallouchos, Maria-Evangelia Filippousi, Georgios Banilas, Aspasia Nisiotou

**Affiliations:** 1 Institute of Technology of Agricultural Products, Hellenic Agricultural Organization “Demeter”, 14123 Lykovryssi, Greece; gsgouros@mbg.duth.gr (G.S.); filippes90mary@hotmail.com (M.-E.F.); 2 Department of Molecular Biology & Genetics, Democritus University of Thrace, 68100 Alexandroupolis, Greece; 3 Laboratory of Food Chemistry and Analysis, Department of Food Science and Human Nutrition, Agricultural University of Athens, 11855 Athens, Greece; aMallouchos@aua.gr; 4 Department of Wine, Vine and Beverage Sciences, University of West Attica, 12243 Athens, Greece; gban@uniwa.gr

**Keywords:** biological acidification, wine fermentation, yeast starter cultures, *Lachancea thermotolerans*, wine chemical profile, volatiles, transcriptional analysis, lactic acid, lactate dehydrogenase, alcohol dehydrogenase

## Abstract

Lactic acid production is an important feature of the yeast *Lachancea thermotolerans* that has gained increasing interest in winemaking. In particular, in light of climate change, the biological acidification and ethanol reduction by the use of selected yeast strains may counteract the effect of global warming in wines. Here, the enological potential of a high lactate-producing *L. thermotolerans* strain (P-HO1) in mixed fermentations with *S. cerevisiae* was examined. Among the different inoculation schemes evaluated, the most successful implantation of *L. thermotolerans* was accomplished by sequential inoculation of *S. cerevisiae*, i.e., at 1% vol. ethanol. P-HO1produced the highest levels of lactic acid ever recorded in mixed fermentations (10.4 g/L), increasing thereby the acidity and reducing ethanol by 1.6% vol. *L. thermotolerans* was also associated with increases in ethyl isobutyrate (strawberry aroma), free SO_2_, organoleptically perceived citric nuances and aftertaste. To start uncovering the molecular mechanisms of lactate biosynthesis in *L. thermotolerans*, the relative expressions of the three lactate dehydrogenase (*LDH*) paralogous genes, which encode the key enzyme for lactate biosynthesis, along with the alcohol dehydrogenase paralogs (*ADH*s) were determined. Present results point to the possible implication of *LDH2*, but not of other *LDH* or *ADH* genes, in the high production of lactic acid in certain strains at the expense of ethanol. Taken together, the important enological features of P-HO1 highlighted here, and potentially of other *L. thermotolerans* strains, indicate its great importance in modern winemaking, particularly in the light of the upcoming climate change and its consequences in the grape/wine system.

## 1. Introduction

Winemaking is principally conducted by the yeast *Saccharomyces cerevisiae*. However, several other yeast species originating from grape berry skins, known as non-*Saccharomyces* or wild yeasts, may evolve during grape must fermentation. Recent studies on the description of yeast community evolution during alcoholic fermentation have revealed several species and strains present mainly at the early- and mid-stages of the course [1,2]. The role of these yeasts seems to be crucial in shaping the flavor and aroma of wines [2,3,4] through the production of important metabolites, including both volatile compounds, like aldehydes, esters or ketones, and non-volatile compounds, like glycerol and lactic acid. *Lachancea thermotolerans* (formerly *Saccharomyces veronae* and then classified as *Kluyveromyces thermotolerans*) is one such wild yeast species with great enological potential, among other important biotechnological applications [5]. Its importance in winemaking relies on a variety of aroma compounds it may confer to wines [6]. Among other metabolites, selected strains of *L. thermotolerans* may produce high levels of lactic acid during alcoholic fermentation, increasing thereby the acidity of wines [7,8,9]. Concentrations may range significantly between 1.0 and 16.8 g/L in *L. thermotolerans* monocultures [9]. In mixed fermentations with *S. cerevisiae*, the lactic acid production may vary from 0.18 to 6.38 g/L, depending on the inoculated strain or fermentation conditions [4,5,8,10]. Recently, a selected strain has been used as commercial starter in mixed dry yeast inocula for wine production [11]. It should be noted, however, that lactic acid in wine is mainly produced from the decarboxylation of L-malic acid by the metabolic activity of lactic acid bacteria (LAB) during malolactic fermentation (MLF). MLF usually follows the alcoholic fermentation, resulting in reduced wine acidity.

Considerable production of lactic acid from *L. thermotolerans* strains was first shown by Whiting [12]. This ability makes *L. thermotolerans* unique among yeasts, as the production of lactic acid by other species is very rare. The major flux of pyruvate metabolism in *S. cerevisiae* is to ethanol, although the fermentation to lactate through lactate dehydrogenase (LDH; E.C. 1.1.1.27) may serve as an alternative route that equally satisfies the re-oxidation of NADH. The molecular mechanisms underlying high yields of lactate production in *L. thermotolerans* at the expense of ethanol or other metabolites are still poorly understood [13]. The genetic basis of high inter-strain variation in lactate productivity is also unknown [9]. Three lactate dehydrogenase genes have been identified in *L. thermotolerans* that are supposed to interconvert pyruvate into lactate with concomitant interconversion of NADH and NAD^+^, yet their relative contributions to lactate biosynthesis is unclear. 

Since the major wine yeast *S. cerevisiae* produces only traces of lactic acid, well-selected *L. thermotolerans* strains could serve ideally for biological acidification of low-acidity grape musts. Such grape musts are common in regions with prolonged high temperatures during summertime, like many places in southern Europe and Australia, and are likely to proliferate due to the upcoming consequences of climate change. Lactic acid is also of great importance for the food, cosmetic, pharmaceutical and biomedical industries. It has been traditionally used as a food preservative, pH-adjusting ingredient or flavoring agent. Hence, the lactic acid market reaches several hundred million dollars annually and a prompt increase is expected in the near future [14,15]. To date, most of the lactate used commercially is produced through lactic acid fermentation of carbohydrates by LAB. However, bacterial lactic acid fermentation has several limitations, such as costly recovery procedures and nutritionally rich media for LAB growth [16,17]. The use of yeasts or fungal strains as lactate producers has the advantage of high tolerance to low pH levels during lactate fermentation, as compared to LAB. However, the yield is still low, while fungal mycelia may block the fermentation [18,19]. Therefore, as an alternative, the genetic manipulation of yeasts, in order to shift the glycolytic flux towards the production of lactic acid, has been attempted through the heterologous expression of different L-lactate dehydrogenase genes. Several strains of *S. cerevisiae* or wild yeast genera, like *Kluyveromyces*, *Candida*, *Pichia*, *Torulaspora* and *Zygosaccharomyces*, have been used. Several limitations still exist, like low yield and inability of cells to efflux lactic acid, which eventually results in LDH inhibition [14,20].

In a previous study, different *L. thermotolerans* strains from vineyards in Greece were isolated and a highly discriminant SSR-based genotyping method was developed [9]. A subpopulation of strains from the island of Crete was shown to produce unusually high levels of lactic acid, reaching 16.6 g/L in monocultures, which is the highest amount recorded so far. In the present study, the behavior of a selected strain of enological potential was investigated during mixed alcoholic fermentations with *S. cerevisiae*, in order to better understand the potential of a high lactate-producing *L. thermotolerans* strain in winemaking. Furthermore, the molecular background of lactic acid production in *L. thermotolerans* was investigated for the first time through transcriptional analysis of key genes in fermentative sugar metabolism.

## 2. Materials and Methods

### 2.1. Yeast Strains

The vineyard-associated *L. thermotolerans* strains used were isolated and identified previously [9]. *S. cerevisiae* PzV6 was isolated from spontaneously-fermenting grape must (Vilana cv., *Vitis vinifera* L.) from the Peza viticultural zone in Crete, Greece. The commercial *S. cerevisiae* strain Flavour 2000 (Enologica Vason S.p.A, Verona, Italy) was also included in the analysis. Yeast strains were cryogenically preserved (−80 °C) and cultured in yeast peptone dextrose agar (20 g/L glucose, 20 g/L peptone, 10 g/L yeast extract, 20 g/L agar) plates. 

### 2.2. Fermentations in Pasteurized Grape Must

Triplicate fermentations were carried out in a pasteurized grape must (70 °C, 10 min) of Vilana cv. from Peza with the following characteristics: sugars 200 g/L; pH 3.42; titratable acidity 5.46 g/L, as tartaric acid; yeast assimilable nitrogen 240 mg/L; total sulphur dioxide 30 mg/L. Fermentations were conducted at 20 °C in 1 L Erlenmeyer flasks containing 750 mL clarified must and supplied with fermentation locks to allow only CO_2_ to escape. Yeast inocula were propagated in grape must at 28 °C for 18 h under shaking (225 rpm). Each yeast strain was added at 6 Log CFU/mL under the following inoculation protocols: indigenous *S. cerevisiae* PzV6 strain (Is); *L. thermotolerans* P-HO1 strain and PzV6 added simultaneously (SmLt); P-HO1 followed by PzV6 after ca. 1% vol. ethanol production (SqLt); and commercial *S. cerevisiae* (Cs). The fermentation course was followed by monitoring the CO_2_ exhaust as measured by weight loss.

### 2.3. Fermentations in Natural Grape Must

Fermentations were conducted in triplicate at 20 °C, in 2.5 L food grade plastic vessels with 2.2 L clarified must of Vilana cv. (sugars 207.6 g/L; pH 3.31; titratable acidity 5.48 g/L, as tartaric acid; yeast assimilable nitrogen 240 mg/L; total sulphur dioxide 30 mg/L). The vessel was closed with a silicone stopper supplied with a muller valve containing glycerol 50% v/v to allow only CO_2_ to escape. Yeast inocula were propagated in grape must at 28 °C for 18 h under shaking (225 rpm). *L. thermotolerans* P-HO1 and *S. cerevisiae* PzV6 were added at 6 log CFU/mL in Is, SmLt and SqLt inoculation schemes, as described in pasteurized must fermentations. Spontaneous fermentations (Sp) were also conducted as reference. The course of the fermentation was monitored by density measurements.

### 2.4. Microbiological Analysis 

Yeast populations were estimated by plate counts, using Wallerstein laboratory nutrient agar (WL; Lab M Bury, Lancashire UK), lysine agar (LA; Lab M Bury, UK) and ethanol sulfite agar (ESA) for the enumerations of total yeasts, non-*Saccharomyces* yeasts and *S. cerevisiae*, respectively. Plates were incubated at 28 °C for 2–5 days. Putative *L. thermotolerans* and *S. cerevisiae* colonies isolated from the initial, middle and final stages of non-sterile fermentations were examined microscopically and subjected to genotyping as previously described [9,21].

### 2.5. Chemical Analysis

Total and volatile acidity, total and free SO_2_, and pH of musts and wines were determined according to the methods in the Compendium of International Methods of Analysis of Musts and Wines [22]. Yeast assimilable nitrogen (YAN) was estimated with the formol titration method as described by Gump et al. [23]. Wine organic acids (citric, tartaric, malic, succinic, lactic and acetic acid), sugars (glucose, fructose), glycerol and ethanol were determined on a JASCO HPLC system (JASCO International Co. Ltd., Tokyo, Japan), consisting of a quaternary gradient pump (PU-2089 plus), an auto sampler (AS-1555) and a refractive index detector (RI-930), as described previously [3]. The major volatile components (i.e., acetaldehyde, ethyl acetate, methanol, 1-propanol, 2-methyl-1-propanol (isobutanol), 3- and 2-methyl-1-butanol) of wines produced from pasteurized grape must were estimated by direct injection of wines in a gas chromatograph (GC 8000 series, model 8060, Fisons Instruments, Milan, Italy) equipped with a split/splitless injector and flame ionization detector, as described by Nisiotou et al. [3]. The volatile compounds of wines produced from natural grape must were determined using a headspace solid-phase microextraction gas chromatography-mass spectrometry (SPME/GC-MS) method [24] with slight modifications [3]. Peaks were quantified using internal standard (IS) calibration curves. If authentic compounds were not available, the concentrations were expressed relatively to IS, i.e., by dividing the peak area of the compound of interest by the peak area of the IS and multiplying this ratio by the concentration of the IS.

### 2.6. RNA Preparation and First-Strand cDNA Synthesis 

Cells were grown in 50 mL filter-sterilized grape must at 28 °C in 250 mL Erlenmeyer flasks under shaking (225 rpm). Cells (2 mL) were collected at early stationary phase (cultivation for 16 h) and centrifuged at 14,000 rpm for 5 min. They were then washed in dd H_2_O, centrifuged again, and resuspended in 150 μL dd H_2_O. To this cellular suspension, 150 μL of a breaking buffer solution (4% v/v Triton X-100, 0.2% w/v SDS, 200 mM NaCl, 20 mM Tris-HCl pH 8.0, 2 mM EDTA) and 300 μL phenol were sequentially added. Eppendorf tubes were then vigorously vortexed for 5 min, incubated at 65 °C for 5 min, vortexed again for 3 min, and centrifuged (14,000 rpm, 8 min). The supernatant was transferred into a new tube, an equal volume of phenol/chloroform (1:1) was added, vortexed and centrifuged as above. The above phenol/chloroform extraction step was repeated solely with chloroform. The aqueous phase was then transferred into a new tube in which 500 μL ice-cold 100% ethanol were added and placed in deep freeze (−80 °C) for 30 min. After centrifugation for 25 min at 4 °C, the pellet was air-dried and diluted in 20 μL dd H_2_O. RNA quality and quantity were assessed using NanoDrop 2000 (Thermo Scientific, Wilmington, DE, USA) and agarose gel electrophoresis. First-strand cDNA was synthesized from about 1 μg of total RNA with a PrimeScript RT Reagent Kit that includes a genomic DNA elimination reaction (Perfect Real Time; Takara, Otsu, Japan), according to the manufacturer’s protocol.

### 2.7. RT-qPCR Analysis

Gene expression analysis was conducted through the reverse transcription quantitative polymerase chain reaction (RT-qPCR). About 15 ng of cDNA was amplified using KAPA SYBR FAST qPCR Master Mix (2×) Kit Real-time RT-PCR (Kapa Biosystems, Woburn, MA, USA) in a StepOnePlus^TM^ (Applied Biosystems, Carlsbad, CA, USA) Real-Time PCR Detection System, according to the manufacturer’s protocol. Amplification conditions consisted of 95 °C for 3 min, followed by 40 cycles of 95 °C for 20 sec, 57 °C for 20 sec, and 72 °C for 10 sec, before melting curve analysis at the end of the run. All reactions were performed in triplicate. The primers used were designed using Primer3web version 4.0 (http://primer3.ut.ee/; part of services provided by ELIXIR - European research infrastructure for biological information). The genes analyzed and their targeted oligonucleotide sequences used are shown in Table 1. *TAF10* was employed as a control housekeeping gene based on the report of Teste et al. [25]. The strain *L. thermotolerans* JCM 19085 (=CBS 6340; type strain) was used as a reference strain. The relative gene expression levels were calculated using the 2^−ΔΔCt^ method.

### 2.8. Sensory Analysis

Twelve experienced assessors (three male and nine female), members of the Institute of Technology of Agricultural Products, Athens, Greece, were recruited. Eleven aroma description terms (estery, apple/pear, orange, lemon, banana, melon, pineapple, floral, citrus, rose and jasmine) and seven taste/flavor terms (fruity flavor, acidity, sweetness, bitterness, viscosity, hotness and after-taste) were developed by the assessors in preliminary sessions. Wines were presented to panelists in a randomized order in duplicate. Each descriptor was scored by marking the perceived intensity on an unstructured 10 cm scale from 0 (absence) to 10 (high intensity).

### 2.9. Statistical Analysis

The chemical and sensory profiles of wines were compared by analysis of variance (ANOVA) and post-hoc Tukey’s HSD test. Chemical parameters were analyzed by principal component analysis (PCA). Different inoculation protocols were compared by permutational multivariate analysis of variance (PERMANOVA). Statistical analyses were performed with PAST software version 3.11 [26] or SPSS v26 (IBM Corp., Armonk, NY, USA).

## 3. Results

### 3.1. Fermentation Kinetics and Yeast Population Dynamics in Pasteurized Grape Must 

Different inoculation scenarios were examined in pasteurized grape must fermentations, i.e., single inoculations with the indigenous strain PzV6 (Is) or the commercial *S. cerevisiae* strain Flavour 2000 (Cs), and mixed inoculations with *L. thermotolerans* P-HO1 and *S. cerevisiae* PzV6, added either simultaneously (SmLt) or sequentially (SqLt). Although PzV6 showed a more vigorous start up to 3.5 days in, it was then outreached by the commercial strain, which finished the fermentation 1.5 days earlier. The two strains also differed significantly in the total amount of CO_2_ released (*p* < 0.001), being 94.45 g for the commercial strain and 91.35 g for PzV6. Almost identical kinetics, with no differences in the amount of CO_2_ released, were observed for the Is and SmLt inoculation schemes, with SmLt being slightly slower after day 3. On the other hand, SqLt fermentations lasted longer than other fermentations by 2–3 days, also producing significantly lower levels of CO_2_ (87.80 g; *p* < 0.001). 

**Figure 1:**
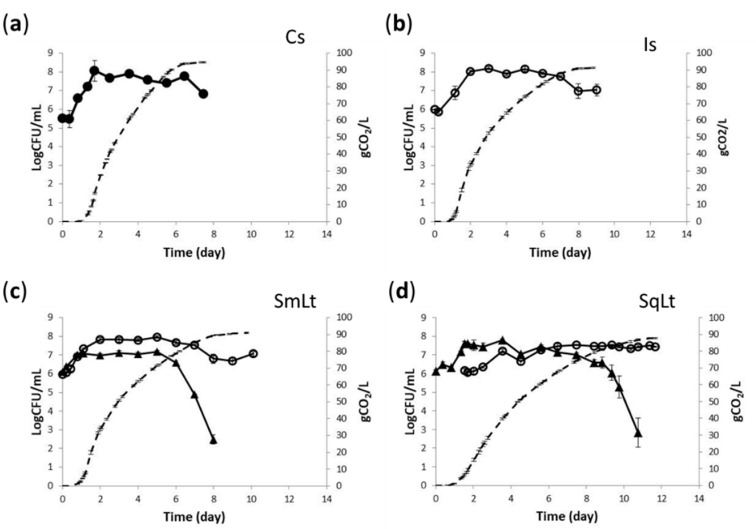
Yeast dynamics (continuous line) of pasteurized must with: (**a**) Cs—commercial *S. cerevisiae* (●); (**b**) Is—indigenous *S. cerevisiae* PzV6 (○); (**c**) SmLt—*L. thermotolerans* P-HO1 (▲) and *S. cerevisiae* PzV6 (○) added simultaneously; and (**d**) SqLt—*L. thermotolerans* P-HO1 (▲) and *S. cerevisiae* PzV6 (○) added sequentially. Dashed line indicates CO_2_ release.

Both *S. cerevisiae* strains showed similar dynamics in single inoculations, reaching maximum populations of 8.20 ± 0.07 and 8.05 ± 0.56 Log CFU/mL for the indigenous and commercial strains, respectively. The strain ScMM23 exhibited identical kinetics in Is and SmLt fermentations (Figure 1). However, PzV6 reached lower population density by ca. 0.67 Log CFU/mL in SqLt compared to Is, which reached maximum populations of 7.53 ± 0.04 Log CFU/mL. On the contrary, P-HO1 counts were higher in SqLt than in SmLt fermentations by 0.62 Log CFU/mL (7.90 ± 0.33 vs. 7.27 ± 0.04 Log CFU/mL, respectively).

### 3.2. Fermentation Kinetics and Yeast Population Dynamics in Natural Grape Must

Is and SmLt exhibited the highest fermentation rates compared to other treatments. Sp showed a rather long lag phase. SqLt had a more dynamic start compared to Sp, but both treatments showed the longest fermentation duration by ca. 3 days compared to all other treatments.

**Figure 2:**
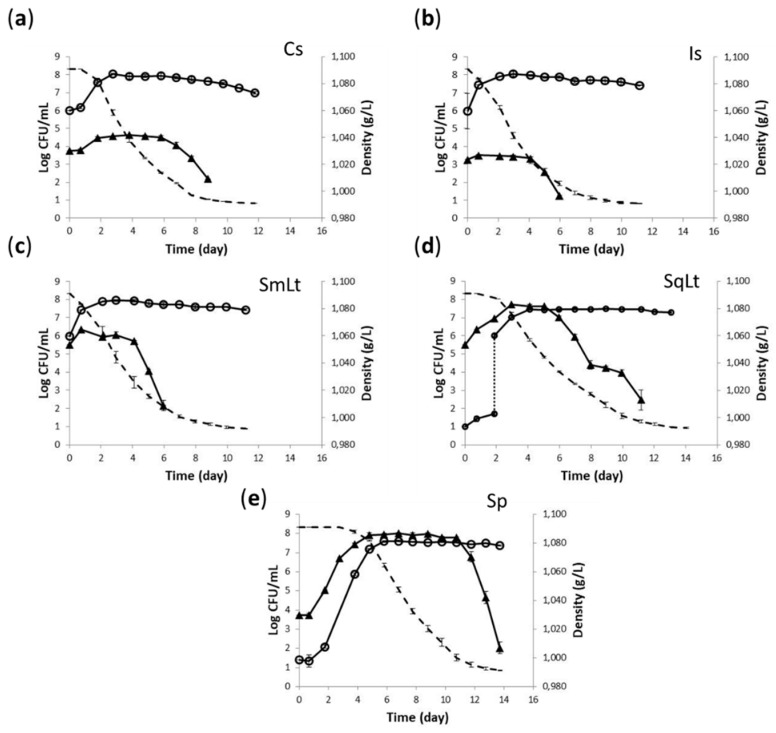
Yeast dynamics (continuous line) of non-sterile must with: (**a**) Cs—commercial *S. cerevisiae*; (**b**) Is—indigenous *S. cerevisiae* PzV6; (**c**) SmLt—*L. thermotolerans* P-HO1 and *S. cerevisiae* PzV6 added simultaneously; (**d**) SqLt—*L. thermotolerans* P-HO1 and *S. cerevisiae* PzV6 added sequentially; and (**e**) Sp—native microbiota. Ethanol sulfite agar was used for enumeration of *S. cerevisiae* (○), and Lysine agar for non-*Saccharomyces* yeasts (▲). Dashed line indicates the density drop during the fermentation course.

Single inoculations with either the commercial *S. cerevisiae* or PzV6 reached maximum cell densities of 8.05 Log CFU/mL. In SmLt, PzV6 showed similar kinetics as in Is, with a maximum population of 7.96 ± 0.06 Log CFU/mL (Figure 2a,b). Counts were lower in SqLt (7.50 ± 0.03 Log CFU/mL) and Sp (7.61 ± 0.05 Log CFU/mL) fermentations (Figure 2c,d) than in other treatments. The dominance of inoculated *S. cerevisiae* strains was confirmed by interdelta PCR fingerprinting at the middle and final stages of fermentations, except for Sp ferment.

Strain P-HO1 showed similar kinetic profiles in natural and pasteurized SqLt fermentations (maximum population of 7.74 ± 0.12 Log CFU/mL and 7.90 ± 0.33 Log CFU/mL, respectively). In SmLt inoculations, counts of P-HO1 were significantly lower in fermentations of natural must than in pasteurized must (maximum population of 6.06 ± 0.05 Log CFU/mL vs. 7.27 ± 0.04 Log CFU/mL, respectively). The addition of *S. cerevisiae* suppressed indigenous non-*Saccharomyces* (NS) yeast populations, which reached maximum populations of only 4.63 ± 0.01 Log CFU/mL and 3.48 ± 0.01 Log CFU/mL in Cs and Is, respectively (Figure 2).

### 3.3. The Effect of Different Inoculation Schemes on the Chemical Profiles of Wines Produced from Pasteurized Grape Must 

**Figure 3:**
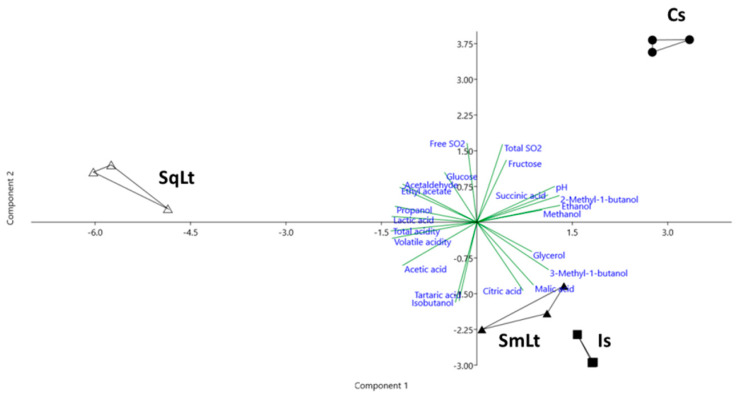
Principal component analysis PCA of the chemical characteristics of wines produced from pasteurized grape must. The first two principal components (PC1 and PC2) accounted for 54.4% and 32.3% of the total variation, respectively. Cs—commercial *S. cerevisiae*; Is—*S. cerevisiae* PzV6; SmLt—*L. thermotolerans* P-HO1 and *S. cerevisiae* PzV6 added simultaneously; SqLt—*L. thermotolerans* P-HO1 and *S. cerevisiae* PzV6 added sequentially.

The chemical characteristics and major volatiles of ferments from pasteurized must are shown in Table 2 and Table 3, respectively. As shown by PERMANOVA, the chemical profile of ferments was significantly affected by the inoculation protocol applied (F = 121.2, *p* = 0.0001). Principal Component Analysis (PCA) was applied to differentiate among the chemical profiles of different ferments (Figure 3). The first two principal components (PC1 and PC2) described 54.4% and 32.3% of the total variability, respectively. Is- and SmLt-derived profiles were grouped closely together, exhibiting high values of malic acid and 3-methyl-1-butanol. Cs formed a distantly separated group along the PC1 and PC2 axes, showing high levels of ethanol and pH. SqLt was placed on the PC1 axis, exhibiting high negative scores for lactic acid and propanol. The means of each individual chemical parameter were compared among the ferments produced by different fermentation protocols. The use of *L. thermotolerans* significantly increased acidity levels. Volatile acidity, lactic acid, acetic acid, ethyl acetate, propanol and acetaldehyde were significantly higher in SqLt compared to other treatments. On the other hand, the ethanol content was significantly reduced. Isobutanol and 3-methyl-1-butanol were increased in Is ferment, while 2-methyl-1-butanol was increased in Cs. 

### 3.4. The Effect of Different Inoculation Schemes on the Chemical Profiles of Wines Produced from Natural Grape Must 

**Figure 4:**
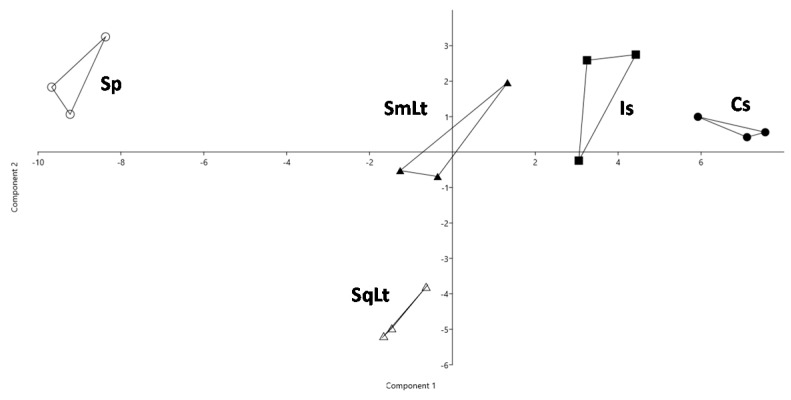
PCA of the chemical characteristics of wines produced from natural grape must. PC1 and PC2 account for 35.7% and 22.4% of the total variation, respectively. Cs—commercial *S. cerevisiae*; Is—*S. cerevisiae* PzV6; SmLt—*L. thermotolerans* P-HO1 and *S. cerevisiae* PzV6 added simultaneously;SqLt—*L. thermotolerans* P-HO1 and *S. cerevisiae* PzV6 added sequentially; Sp—spontaneous fermentation.

The chemical characteristics and volatile metabolites of wines produced in natural musts are presented in Table 4 and Table 5, respectively. PERMANOVA revealed significant discrepancies in the chemical profiles of wines produced through different inoculation protocols (F = 24.2, *p* = 0.0001). Differences in the chemical profiles were visualized by PCA (Figure 4). PC1 and PC2 accounted for 64.5% and 14.9%, respectively, of total variability. SqLt was negatively differentiated on PC2 for several attributes, such as total acidity, lactic acid and phenylethyl alcohol. Both Cs and Is were placed on the positive quadrant, showing highly positive scores on PC1 for 3-methylbutyl acetate, 2-phenylethyl acetate and hexyl acetate. SmLt was placed between Is and SqLt, while Sp was the most distantly located group among all ferments.

Means of chemical attributes were compared by ANOVA. Total acidity was significantly higher (*p* < 0.05) in SqLt than in other treatments (Table 4). The addition of P-HO1 in SmLt or SqLt did not increase the volatile acidity compared to Is. The ethanol content was significantly lower in SqLt compared to other inoculation schemes. Increased levels of ethyl isobutyrate, 1-butanol, ethyl lactate, 1-hexanol and 2-methylpropanoic acid, and reduced levels of acetaldehyde, isobutyl acetate, 3-methylbutyl acetate, ethyl hexanoate, ethyl octanoate, acetic acid, butanoic acid 2-phenylethyl acetate and octanoic acid, were observed in SqLt.

### 3.5. Transcriptional Analysis of L. thermotolerans LDH and ADH Genes

**Figure 5:**
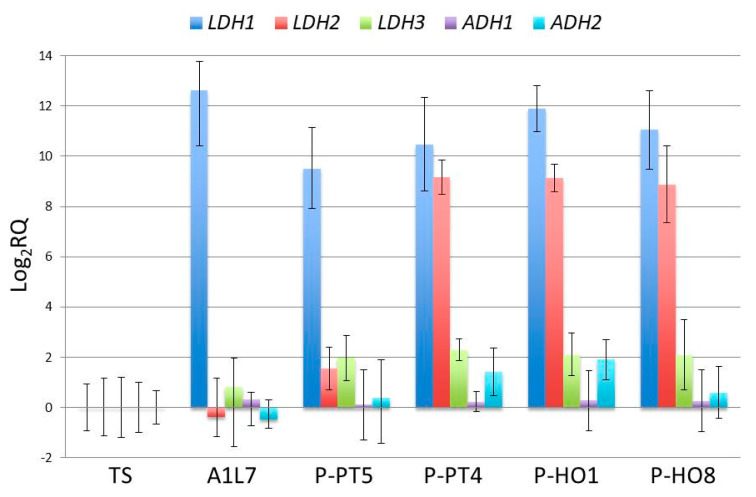
Quantitative PCR (qPCR) analysis of the *L. thermotolerans* lactate dehydrogenase (*LDH*) and alcohol dehydrogenase (*ADH*) genes in high- (P-PT4, P-HO1, P-HO8) and low- (A1L7, P-PT5) lactic acid-producing strains. The low lactic acid-producing *L. thermotolerans* type strain (TS) was used as reference (Log_2_RQ = 0). RQ—relative quantification. Error bars—log_2_RQmin and log_2_RQmax; RQmin = 2 − (RQ − SE) and RQmax = 2 − (RQ + SE), where SE is the standard error for the RQ.

In order to shed some light in the molecular mechanisms of lactate biosynthesis in *L. thermotolerans*, the relative expressions of three *LDH* genes encoding lactate dehydrogenase, the key enzyme implicated in lactate biosynthesis, were evaluated in vineyard-associated *L. thermotolerans* strains producing high levels of lactate (strains P-HO1, P-PT4 and P-PHO8, producing 15.6 g/L, 14.8 g/L and 10.7 g/L, respectively) or low levels of lactate (strains A1L7 and P-PT5 producing 0.6 g/L and 1.4 g/L, respectively). The relative gene expression was estimated in respect to the type strain *L. thermotolerans*, which produced low amounts of lactic acid (0.5 g/L). Substantial differences were observed in the expression levels of the three *LDH*s (Figure 5). The *LDH1* gene was highly expressed in all vineyard-strains, while *LDH3* was consistently expressed at rather low levels in all strains, comparable to those of the type strain. It is thus plausible that the differential production of lactic acid may not be controlled through the transcriptional regulation of these genes. In contrast, the *LDH2* gene was up-regulated in the high lactate-producing strains, but not in the low lactate-producing strains. In order to investigate whether the high production of lactate might be also related to down-regulation of alcohol dehydrogenase genes (*ADHs*), the enzyme responsible for converting acetaldehyde into ethanol during alcoholic fermentation, both the *L. thermotolerans ADH* genes were included in the comparative transcriptional analysis. As is shown in Figure 5, the expression levels of both *ADH1* and *ADH2* were similar to those of the type strain, and no substantial differences were observed between low lactate-producing and high lactate-producing strains. Taken together, *LDH2* was the only gene in the present analysis that had transcript levels that coincided with the levels of lactic acid production in different strains.

### 3.6. Sensory Analysis

**Figure 6:**
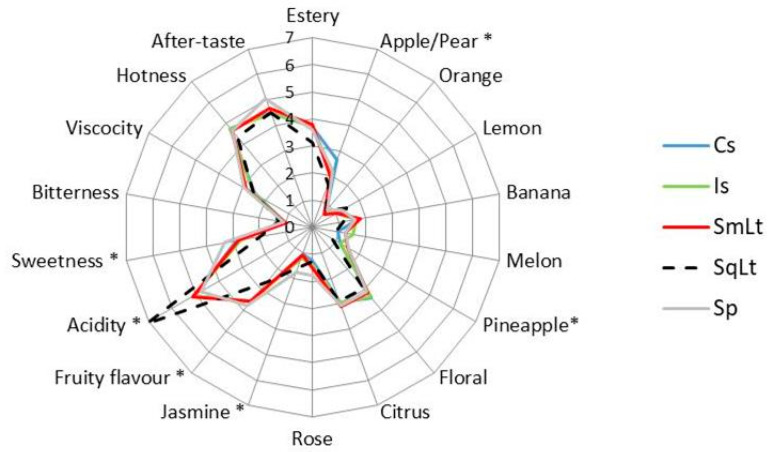
Sensory attributes scores for the wines produced through different inoculation schemes. Cs—commercial *S. cerevisiae*; Is—*S. cerevisiae* PzV6; SmLt—*L. thermotolerans* P-HO1 and *S. cerevisiae* PzV6 added simultaneously; SqLt—*L. thermotolerans* P-HO1 and *S. cerevisiae* PzV6 added sequentially; Sp—spontaneous fermentation. Asterisk indicates significant differences among samples (*p* < 0.05).

The average scores assigned by the panel for each attribute of Vilana cv. wines are shown in Figure 6. Six attributes were found to differ significantly (*p* < 0.05) among wines, i.e., apple/pear, pineapple, jasmine, fruit flavor, acidity and sweetness. Acidity was significantly higher in SqLt than other treatments, followed by SmLt. Sp showed significantly higher sweetness, fruit flavor and jasmine or pineapple aromas than other wines. Jasmine and pineapple aromas were also highly rated in SqLT and SmLt, respectively. Cs-derived wine was characterized by high apple/pear aroma. Compared to other treatments, SqLt was characterized by higher orange and lemon nuances, reduced hotness and good aftertaste.

## 4. Discussion

Lactic acid production is an important feature of *L. thermotolerans* metabolism that has gained increasing interest in enology in light of climate change. Global warming, in particular, is expected to have a significant impact on wine quality, by reducing acidity and increasing ethanol content. Therefore, biological acidification, and the development of ethanol reduction procedures by the use of appropriate yeast species/strains, could offer an advantageous alternative in modern winemaking, especially in warm viticultural regions. Despite the high importance of lactate biosynthesis in *L. thermotolerans* for winemaking and also for various other biotechnological applications, the performance of high lactic acid-producing strains in wine fermentation has not been studied in depth, and the respective molecular mechanism underlying the unusually high levels of lactic acid production in this species is largely unknown [13]. Accumulating data show that *L. thermotolerans* isolated from vineyards or other ecological niches may exhibit significantly different metabolic activities [9,13]. There is considerable diversity among strains in the production of various metabolites, including ethanol, acetic acid, esters, terpens and lactic acid or other secondary metabolites of alcoholic fermentations.

Here, a high lactate-producing *L. thermotolerans* strain (P-HO1) that had been previously identified [9] was further characterized, and its enological potential in mixed fermentations with *S. cerevisiae* was evaluated. As expected, in simultaneous inoculations the P-HO1 population was suppressed by *S. cerevisiae*, probably due to the highly antagonistic features of *S. cerevisiae* in grape must fermentations (e.g., high sugar fermentation capacity and nitrogen uptake) [27,28]. As a result, the metabolic contribution of P-HO1 in SmLt ferment was limited. Contrastingly, in sequential inoculations with *S. cerevisiae* (SqLt), P-HO1 was highly active and capable of producing high levels of lactic acid. Notably, lactic acid levels exceeded 10 g/L in SqLt, which, to our knowledge, is the highest amount ever recorded in mixed-inoculated alcoholic fermentations. Subsequently, the total acidity was increased by 9.2 g/L, which is also the highest rise ever observed. Ethanol content declined greatly, by 1.6% vol. in sterile and 0.6% vol. in natural grape must. Such reduction may counterbalance the average increase in alcohol content of wines in warmer regions, which has been estimated to be approximately 2% over the last 30 years [29].

Strain P-HO1 produced significant levels of lactic acid during fermentation of natural grape must, albeit lower than in pasteurized must. This might be due to the lower prevalence of *L. thermotolerans* in natural than in pasteurized must, since the production of lactic acid depends on the concentration of yeast cells [6]. In addition, *L. thermotolerans* has been shown to exhibit limited maximum population when low oxygen is present [8]. Taken together, the above features indicate that strain P-HO1 could serve as a promising alternative for efficient acidification and concurrent ethanol reduction in winemaking, offering to modern winemakers an alternative for producing balanced wines with non-invasive chemical or physicochemical means.

P-HO1 was shown to lower the levels of esters in SqLt, in accordance with previous observations [8,30,31,32]. However, here it was also noticed that the reduction was associated with increases in ethyl isobutyrate (strawberry aroma), as well as organoleptically perceived citric (lemon and orange) and jasmine nuances. The use of *L. thermotolerans* was also accompanied by some more interesting organoleptic characteristics, such as decreased hotness and good aftertaste. Another positive effect of acidification was the increase in the levels of free SO_2_, facilitating thereby a reduction in total SO_2_ supplementation. Our data show that the successful implantation of *L. thermotolerans* is accomplished by late sequential inoculation of *S. cerevisiae*, i.e., after ca. 1% vol. ethanol production, corroborating previous results [6,8]. By this means the acidification of fermented must is expedited, since lactic acid is produced at the early stages of fermentation [5].

**Figure 7:**
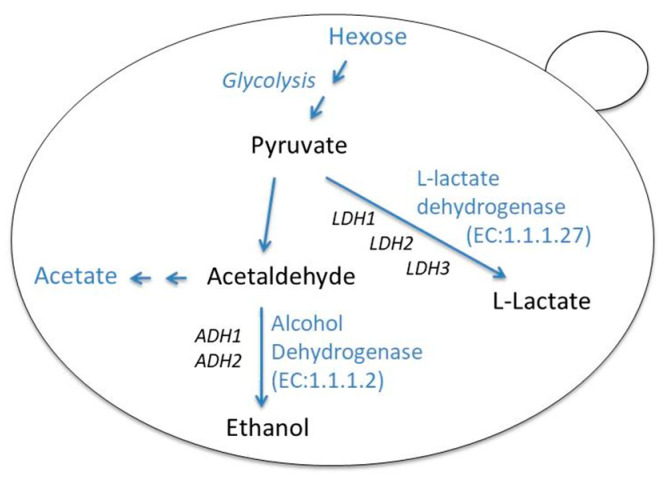
Simplified schematic representation of fermentative sugar metabolism in *L. thermotolerans* based on KEGG (Kyoto Encyclopedia of Genes and Genomes) Pathway Database; https://www.genome.jp/dbget-bin/www_bget?lth00010. Pyruvate can be directly converted into lactate through the action of L-lactate dehydrogenase (LDH) or into ethanol by alcohol dehydrogenase (ADH). In the genome of *L. thermotolerans* there are 3 paralogs encoding LDH and 2 *ADH* paralogous genes.

Although the production of lactic acid is a key metabolic characteristic of *L. thermotolerans*, there is substantial heterogeneity among strains in the amount of lactic acid they produce [9]. During fermentative sugar metabolism in *L. thermotolerans*, ethanol and lactate are the predominant products along with acetate, which is excreted in small amounts with minimal inter-strain variation [9,13]. Thus, the most important steps in pyruvate metabolism of *L. thermotolerans* during grape must fermentation seem to be the conversion of pyruvate into lactate by the activity of lactate dehydrogenase (LDH), and to ethanol by the function of alcohol dehydrogenase (ADH) (Figure 7). Based on the whole genome sequence of the type strain [33], *L. thermotolerans* possesses three *LDH* (*LDH1*, *LDH2* and *LDH3*) and two *ADH* (*ADH1* and *ADH2*) paralogous genes. Here we asked whether the high production of lactate in strain P-HO1, and other strains as well, might be attributed to up-regulation of *LDH* and/or down-regulation of *ADH*s genes. Comparative transcriptional analysis in high and low lactate-producing strains revealed a potential role of *LDH2*, but not of the other genes. Another interesting observation made was the differential regulation of the three *LDH* paralogs. It would be important to see whether the expression differences of these genes are coupled with functional differences, as in the case of the *S. cerevisiae ADH* genes [34,35].

Present results show that the use of the vineyard *L. thermotolerans* P-HO1 strain in mixed fermentations with *S. cerevisiae* PzV6, especially when inoculated in a sequential mode, may serve as a powerful and useful tool for reducing the alcohol content of wines through the production of lactic acid, thus leading to simultaneous biological acidification. The molecular mechanism underlying the unusually high production of lactic acid indicates the possible involvement of *LDH2*, rather than other *LDH* paralogs or *ADH* genes. Apart from several other biotechnological applications, the important features of P-HO1 and potentially of other *L. thermotolerans* strains are expected to be of high value for the wine industry. Based on the present results, future research on the genetic manipulation of yeasts for the overproduction of lactic acid could also consider the use of selected *L. thermotolerans* strains, or the heterologous expression of the *L. thermotolerans LDH2* gene.

## Figures and Tables

**Table 1:** Genes and primers used for quantitative RT-PCR analysis. KEGG GENES database is available at http://www.genome.jp/kegg/genes.htmL (Kyoto University Bioinformatics Center, Kyoto, Japan).

KEGG Entry/ Gene Name	Protein [KEGG ENZYME]	Forward Primer/ Reverse Primer (5′–3′)
KLTH0D00440g/*LDH1*	L-lactate dehydrogenase [EC:1.1.1.27]	ATTATCACAGGAGGAGCCAATC/ AGAAGCAAAATGGTGTCAGGAG
KLTH0G19536g/*LDH2*		ATCTACCCACCAGCCTCAAC/ TTTCGCCCTCTGCTTTATGT
KLTH0G19558g/*LDH3*		GCAGAGTTATCGGCTCAGGTA/ TGGCTTTTTCGCAGTAATCC
KLTH0B04972g/*ADH1*	Alcohol dehydrogenase [EC:1.1.1.2]	GGTGTCTTCGTGCTTGGACT/ TAGGTGCGTTGGGATTTTCT
KLTH0E07964g/*ADH2*		GCTTCCCTATCCCACCAGAG/ ACGCTTGGAGTAACCACGAG
KLTH0G11352g/*TAF10*	Transcription initiation factor TFIID subunit 10	CACGGTTCACAACTCCAACA/ CGCTGCTTAGGTCGTTCACT

**Table 2:** Chemical characteristics of wines produced from pasteurized grape must (mean ± SD, *n* = 3). Values with different superscript letters within each row differ significantly (*p* < 0.05).

Chemical Component	Inoculation Protocol ^1^			
	Cs	Is	SmLt	SqLt
Total acidity (as tartaric acid g/L)	6.3 ± 0.1 ^d^	7.8 ± 0.1 ^c^	9.2 ± 0.1 ^b^	15.5 ± 0.1 ^a^
pH	3.53 ± 0.01 ^a^	3.38 ± 0.01 ^b^	3.38 ± 0.01 ^b^	3.24 ± 0.01 ^c^
Volatile acidity (as acetic acid g/L)	0.11 ±0.01 ^c^	0.16 ±0.00 ^b^	0.15 ±0.01 ^b^	0.28 ±0.02 ^a^
Free SO_2_ (mg/L)	10.0 ± 0.0 ^a^	6.8 ± 0.8 ^b^	7.3 ± 0.8 ^b^	9.0 ± 0.0 ^a^
Total SO_2_ (mg/L)	22.2 ± 0.7 ^a^	16.3 ± 0.6 ^b^	17.5 ± 1.5 ^b^	18.0 ± 0.1 ^b^
Citric acid (mg/L)	387 ± 3 ^c^	446 ± 3 ^a^	418 ± 9 ^b^	377 ± 10 ^c^
Tartaric acid (g/L)	1.0 ± 0.1 ^c^	1.3 ± 0.1 ^a^	1.2 ± 0.1 ^a^	1.2 ± 0.0 ^a,b^
Malic acid (g/L)	1.9 ± 0.0 ^c^	2.6 ± 0.0 ^a^	2.3 ± 0.1 ^b^	1.6 ± 0.0 ^d^
Glucose (g/L)	<0.6	<0.6	<0.6	<0.6
Fructose (g/L)	1.0 ± 0.0	<0.6	<0.6	<0.6
Succinic acid (g/L)	1.2 ± 0.0 ^a^	1.1 ± 0.0 ^b^	1.2 ± 0.0 ^a^	1.0 ± 0.0 ^c^
Lactic acid (g/L)	< 0.6	< 0.6	2.3 ± 0.1 ^b^	10.4 ± 0.3 ^a^
Glycerol (g/L)	6.9 ± 0.1 ^a^	7.0 ± 0.0 ^a^	7.2 ± 0.2 ^a^	6.7 ± 0.2 ^b^
Acetic acid (mg/L)	< 10	68 ± 2 ^c^	80 ± 5 ^b^	193 ± 1 ^a^
Ethanol (g/L)	99.4 ± 0.9 ^a^	96.3 ± 0.4 ^a^	93.0 ± 0.3 ^b^	87.0 ± 1.6 ^c^

^1^ Cs—commercial *S. cerevisiae*; Is—*S. cerevisiae* PzV6; SmLt—*L. thermotolerans* P-HO1 and *S. cerevisiae* PzV6 added simultaneously; SqLt—*L. thermotolerans* P-HO1 and *S. cerevisiae* PzV6 added sequentially; SD – Standard Deviation.

**Table 3:** Major volatiles (mg/L) of wines produced from pasteurized grape must (mean ± SD, *n* = 3). Values with different superscript letters within each row differ significantly (*p* < 0.05).

Chemical Component	Inoculation Protocol ^1^			
	Cs	Is	SmLt	SqLt
Acetaldehyde	7.5 ± 0.5 ^b^	6.4 ± 0.7 ^b^	6.3 ± 0.3 ^b^	11.0 ± 1.2 ^a^
Ethyl acetate	35.8 ± 2.0 ^b^	29.8 ± 0.9 ^c^	32.3 ± 1.1 ^b^	54.4 ± 1.6 ^a^
Methanol	46.9 ± 2.5 ^a^	45.7 ± 1.7 ^a^	43.8 ± 2.9 ^a^	41.6 ± 2.4 ^a^
Propanol	26.2 ± 1.7 ^b^	24.4 ± 0.5 ^c^	28.8 ± 0.2 ^b^	39.5 ± 1.3 ^a^
Isobutanol	40.2 ± 2.5 ^c^	69.1 ± 2.0 ^a^	63.4 ± 1.8 ^b^	58.9 ± 2.0 ^b^
2-Methyl-1-butanol	92.8 ± 6.1 ^a^	71.7 ± 1.3 ^b^	66.6 ± 2.1 ^b^	44.4 ± 1.1 ^c^
3-Methyl-1-butanol	233.2 ± 14.6 ^c^	306.7 ± 9.3 ^a^	278.8 ± 6.8 ^b^	153.4 ± 4.1 ^d^

^1^ Cs—commercial *S. cerevisiae*; Is—*S. cerevisiae* PzV6; SmLt—*L. thermotolerans* P-HO1 and *S. cerevisiae* PzV6 added simultaneously; SqLt—*L. thermotolerans* P-HO1 and *S. cerevisiae* PzV6 added sequentially.

**Table 4:** Chemical characteristics of wines produced from natural grape must (mean ± SD, *n* = 3). Values with different superscript letters within each row differ significantly (*p* < 0.05).

Chemical Component	Inoculation Protocol ^1^				
	Cs	Is	SmLt	SqLt	Sp
Total acidity (as tartaric acid g/L)	6.2 ± 0.1 ^b,c^	6.1± 0.0 ^c^	6.1 ± 0.1 ^b,c^	10.2 ± 0.3 ^a^	6.5 ± 0.1 ^b^
pH	3.34 ± 0.01 ^a^	3.29 ± 0.01 ^b^	3.27 ± 0.01 ^b^	3.15 ± 0.01 ^c^	3.32 ± 0.01 ^a^
Volatile acidity (as acetic acid g/L)	0.13 ±0.05 ^c^	0.35 ±0.04 ^b^	0.37 ±0.02 ^b^	0.31 ±0.04 ^b^	0.59 ± 0.04 ^a^
Free SO_2_ (mg/L)	15.8 ± 1.5 ^a^	12.8 ± 0.0 ^a^	14.1 ± 1.3 ^a^	15.8 ± 0.7 ^a^	14.5 ± 1.5 ^a^
Total SO_2_ (mg/L)	24.3 ± 1.3 ^c^	29.9 ± 1.5 ^a^	29.9 ± 0.7 ^a^	29.4 ± 1.3 ^b^	25.2 ± 2.7 ^b,c^
Citric acid (mg/L)	668 ± 3 ^a^	657 ± 10 ^a^	651 ± 4 ^a^	621 ± 4 ^b^	664 ± 9 ^a^
Tartaric acid (g/L)	1.3 ± 0.2 ^b^	1.9 ± 0.2 ^a^	1.8 ± 0.2 ^a^	1.6 ± 0.1 ^a,b^	1.3 ± 0.0 ^b^
Malic acid (g/L)	2.1 ± 0.0 ^a^	2.1 ± 0.1 ^a,b^	2.0 ± 0.0 ^b^	1.8 ± 0.0 ^d^	1.9 ± 0.0 ^c^
Glucose (g/L)	<0.6	<0.6	<0.6	<0.6	<0.6
Fructose (g/L)	1.0 ± 0.1 ^a^	0.9 ± 0.3 ^a^	0.9 ± 0.3 ^a^	1.3 ± 0.3 ^a^	1.0 ± 0.6 ^a^
Succinic acid (g/L)	0.7 ± 0.0 ^a^	<0.6	<0.6	0.6± 0.0 ^a^	<0.6
Lactic acid (g/L)	< 0.6	< 0.6	< 0.6	5.5 ± 0.4	< 0.6
Glycerol (g/L)	6.6 ± 0.0 ^b^	5.7 ± 0.1 ^c^	5.7 ± 0.1 ^c^	6.7 ± 0.1 ^b^	8.3 ± 0.2 ^a^
Acetic acid (mg/L)	299 ± 10 ^c^	525 ± 22 ^b^	522 ± 8 ^b^	522 ± 18 ^b^	737 ± 10 ^a^
Ethanol (g/L)	104.7 ± 0.3 ^b,c^	106.7 ± 0.9 ^a^	106.0 ± 0.6 ^a,b^	102.2 ± 0.5 ^d^	104.2 ± 0.3 ^c^

^1^ Cs—commercial *S. cerevisiae*; Is—*S. cerevisiae* PzV6; SmLt—*L. thermotolerans* P-HO1 and *S. cerevisiae* PzV6 added simultaneously; SqLt—*L. thermotolerans* P-HO1 and *S. cerevisiae* PzV6 added sequentially; Sp—spontaneous fermentation.

**Table 5:** Content of volatile compounds ^1^ in wines produced from natural grape must (mean ± SD, *n* = 3). Values with different superscript letters within each row differ significantly (*p* < 0.05).

Compound ^2^	Inoculation Protocol ^3^				
	Cs	Is	SmLt	SqLt	Sp
*Esters*					
Ethyl acetate (mg/L)	90.3 ± 5.3 ^a^	90.9 ± 12.6 ^a^	86.2 ± 31.4 ^a^	87.0 ± 14.9 ^a^	62.6 ± 4.5 ^a^
Isobutyl acetate	130 ± 13 ^a,b^	167 ± 28 ^a^	137 ± 19 ^a,b^	101 ± 16 ^b^	45 ± 4 ^c^
3-Methylbutyl acetate (mg/L)	5.8 ± 0.5 ^a^	5.5 ± 0.3 ^a^	3.8 ± 0.4 ^b^	3.3 ± 0.6 ^b^	0.5 ± 0.4 ^c^
Hexyl acetate	235 ± 15 ^a^	212 ± 15 ^a^	157 ± 17 ^b^	107 ± 6 ^c^	83 ± 3 ^c^
2-Phenylethyl acetate (mg/L)	1.9 ± 0.1 ^a,b^	2.0 ± 0.1 ^a^	1.5 ± 0.3 ^b^	0.6 ± 0.1 ^c^	0.3 ± 0.0 ^c^
Diethyl succinate	41 ± 1 ^b^	59 ± 12 ^a,b^	104 ± 48 ^a^	47 ± 5 ^a,b^	66 ± 6 ^a,b^
Propyl acetate	88 ± 11 ^a^	70 ± 5 ^a,b^	53 ± 8 ^b^	68 ± 9 ^a,b^	26 ± 2 ^c^
Butyl acetate	24 ± 3 ^a^	18 ± 2 ^b^	14 ± 2 ^b^	18 ± 3 ^a,b^	7 ± 1 ^c^
(3Z)-3-Hexenyl acetate	737 ± 53 ^a^	741 ± 59 ^a^	495 ± 73 ^b^	149 ± 31 ^c^	55 ± 7 ^c^
Ethyl isobutyrate	93 ± 3 ^a,b^	92 ± 2 ^b^	90 ± 2 ^b^	101 ± 5 ^a^	96 ± 2 ^a,b^
Ethyl butanoate	578 ± 46 ^a,b^	609 ± 21 ^a^	487 ± 37 ^b,c^	375 ± 34 ^d^	472 ± 34 ^c^
Ethyl hexanoate (mg/L)	1.1 ± 0.1 ^a^	0.9 ± 0.1 ^a,b^	0.8 ± 0.1 ^b^	0.4 ± 0.1 ^c^	0.9 ± 0.1 ^a,b^
Ethyl octanoate (mg/L)	1.6 ± 0.1 ^a^	1.5 ± 0.3 ^a^	1.3 ± 0.2 ^a^	0.8 ± 0.1 ^b^	1.4 ± 0.2 ^a^
Ethyl dec-9-enoate (mg/L)	1.7 ± 0.2 ^a^	4.1 ± 1.7 ^a^	4.0 ± 1.1 ^a^	2.4 ± 0.1 ^a^	3.8 ± 0.8 ^a^
Ethyl decanoate	938 ± 174 ^a^	929 ± 192 ^a^	823 ± 265 ^a^	767 ± 37 ^a^	938 ± 153 ^a^
Ethyl lactate (mg/L)	2.2 ± 0.2 ^b^	2.1 ± 0.3 ^b^	3.9 ± 1.0 ^b^	112.4 ± 19.0 ^a^	1.3 ± 0.1 ^b^
Isopentyl hexanoate	35 ± 2 ^a^	26 ± 8 ^a,b^	21 ± 5 ^b^	20 ± 2 ^b^	19 ± 4 ^b^
Methyl hexanoate	58 ± 8 ^a^	42 ± 3 ^b,c^	33 ± 4 ^b,c^	31 ± 6 ^c^	45 ± 4 ^a,b^
3-Methylbutyl octanoate	281 ± 78 ^a^	279 ± 67 ^a^	235 ± 67 ^a^	162 ± 18 ^a^	163 ± 30 ^a^
Ethyl dodecanoate (mg/L)	5.5 ± 2.3 ^a^	4.4 ± 1.2 ^a^	5.1 ± 2.1 ^a^	3.1 ± 0.3 ^a^	6.0 ± 0.8 ^a^
*Alcohols*					
1-Propanol (mg/L)	40.9 ± 3.5 ^a,b^	30.7 ± 4.7 ^a,b^	29.2 ± 7.2 ^b^	44.1 ± 7.4 ^a^	40.1 ± 0.5 ^a,b^
2-Methyl-1-propanol (mg/L)	33.0 ± 2.9 ^a^	55.8 ± 23.2 ^a^	52.5 ± 12.8 ^a^	52.8 ± 10.0 ^a^	32.6 ± 1.8 ^a^
3-Methyl-1-butanol (mg/L)	159.2 ± 15.7 ^a,b^	182.8 ± 18.9 ^a^	123.4 ± 29.0 ^b,c^	146.2 ± 16.0 ^a,b^	92.8 ± 9.4 ^c^
1-Butanol	19 ± 1 ^b^	12 ± 1 ^c^	12 ± 3 ^c^	29 ± 4 ^a^	9 ± 1 ^c^
1-Hexanol	318 ± 21 ^b^	317 ± 19 ^b^	379 ± 30 ^b^	553 ± 19 ^a^	545 ± 45 ^a^
(Z)-3-Hexen-1-ol	437 ± 29 ^a^	462 ± 34 ^a^	466 ± 40 ^a^	490 ± 8 ^a^	497 ± 35 ^a^
2-Ethyl-1-hexanol	55 ± 2 ^a^	53 ± 1 ^a^	53 ± 1 ^a^	53 ± 1 ^a^	52 ± 0 ^a^
2,3-Butanediol	102 ± 18 ^a^	67 ± 18 ^a^	82 ± 36 ^a^	60 ± 12 ^a^	91 ± 5 ^a^
2-Phenylethanol (mg/L)	50.0 ± 4.9 ^a^	43.5 ± 3.8 ^a,b^	37.7 ± 5.4 ^b^	38.0 ± 2.3 ^b^	22.1 ± 1.8 ^c^
1-Nonanol	24 ± 2 ^a^	14 ± 1 ^b^	13 ± 4 ^b^	8 ± 0 ^b^	12 ± 3 ^b^
*Acids*					
Acetic acid (mg/L)	1.2 ± 0.5 ^b^	4.4 ± 2.3 ^ab^	2.6 ± 0.2 ^b^	2.3 ± 0.4 ^b^	7.2 ± 1.5 ^a^
2-Methylpropanoic acid	162 ± 36 ^a,b^	173 ± 66 ^ab^	129 ± 22 ^b^	252 ± 17 ^a^	177 ± 9 ^a,b^
Butanoic acid	116 ± 37 ^a,b,c^	162 ± 17 ^a^	144 ± 11 ^a,b^	70 ± 17 ^c^	93 ± 27 ^b,c^
3-Methylbutanoic acid	114 ± 45 ^a^	92 ± 17 ^a^	63 ± 9 ^a,b^	63 ± 4 ^a,b^	26 ± 2 ^b^
2-Methylbutanoic acid	56 ± 16 ^a^	38 ± 7 ^a,b,c^	31 ± 7 ^b,c^	43 ± 2 ^a,b^	19 ± 3 ^c^
Hexanoic acid (mg/L)	1.0 ± 0.3 ^c^	3.0 ± 0.2 ^a^	2.7 ± 0.1 ^a,b^	1.6 ± 0.3 ^c^	2.4 ± 0.2 ^b^
Octanoic acid (mg/L)	0.5 ± 0.3 ^d^	3.6 ± 0.4 ^a^	3.1 ± 0.4 ^a,b^	2.0 ± 0.3 ^c^	2.4 ± 0.2 ^b,c^
Decanoic acid	50 ± 30 ^c^	854 ± 187 ^a^	629 ± 156 ^a,b^	898 ± 70 ^a^	482 ± 48 ^b^
*Terpenes*					
α-Phellandrene	47 ± 16 ^a^	40 ± 15 ^a^	36 ± 6 ^a^	31 ± 7 ^a^	47 ± 8 ^a^
β-Myrcene	34 ± 11 ^a^	28 ± 10 ^a^	25 ± 4 ^a^	22 ± 5 ^a^	34 ± 5 ^a^
D-Limonene	42 ± 8 ^a^	29 ± 11 ^a,b^	24 ± 3 ^a,b^	22 ± 4 ^b^	33 ± 5 ^a,b^
α-Pinene	44 ± 1 ^a^	39 ± 1 ^b^	38 ± 0 ^b^	38 ± 0 ^b^	39 ± 1 ^b^
γ-Terpinene	36 ± 3 ^a^	24 ± 2 ^b^	23 ± 0 ^b^	22 ± 1 ^b^	23 ± 0 ^b^
p-Cymene	35 ± 4 ^a^	17 ± 4 ^b^	14 ± 0 ^b^	14 ± 0 ^b^	15 ± 0 ^b^
Linalool	62 ± 2 ^a^	59 ± 0 ^b^	58 ± 1 ^b^	60 ± 0 ^a,b^	60 ± 1 ^a,b^
α-Terpineol	23 ± 4 ^a^	15 ± 5 ^ab^	11 ± 3 ^b^	13 ± 2 ^a,b^	12 ± 3 ^b^
β-Citronellol	14 ± 6 ^a^	3 ± 1 ^b^	3 ± 0 ^b^	3 ± 0 ^b^	9 ± 1 ^a,b^
β-Damascenone	72 ± 4 ^a^	77 ± 11 ^a^	71 ± 15 ^a^	97 ± 11 ^a^	76 ± 7 ^a^
*Miscellaneous*					
Acetaldehyde	24 ± 2 ^c^	72 ± 14 ^a^	68 ± 18 ^a,b^	40 ± 7 ^b,c^	18 ± 2 ^c^
1,1-Diethoxy-ethane	26 ± 3 ^a^	14 ± 1 ^b^	14 ± 4 ^b^	18 ± 4 ^a,b^	16 ± 1 ^b^
2,3-Butanedione	12 ± 1 ^a^	12 ± 2 ^a^	8 ± 1 ^a,b^	7 ± 1 ^b^	10 ± 1 ^a,b^
2-Octanone	191 ± 18 ^a^	180 ± 23 ^a^	127 ± 2 ^b^	145 ± 17 ^a,b^	166 ± 22 ^a,b^

^1^ If not stated, unit of concentration is μg/L. ^2^ Compounds in gray were determined semi-quantitatively relative to IS. All other compounds were determined using internal standard calibration curves. ^3^ Cs—commercial *S. cerevisiae*; Is—*S. cerevisiae* PzV6; SmLt—*L. thermotolerans* P-HO1 and *S. cerevisiae* PzV6 added simultaneously; SqLt—*L. thermotolerans* P-HO1 and *S. cerevisiae* PzV6 added sequentially; Sp—spontaneous fermentation.
